# Monoclonal Antibodies Specific for STAT3β Reveal Its Contribution to Constitutive STAT3 Phosphorylation in Breast Cancer

**DOI:** 10.3390/cancers6042012

**Published:** 2014-09-29

**Authors:** Uddalak Bharadwaj, Moses M. Kasembeli, T. Kris Eckols, Mikhail Kolosov, Paul Lang, Kurt Christensen, Dean P. Edwards, David J. Tweardy

**Affiliations:** 1 Section of Infectious Disease, Department of Medicine, Baylor College of Medicine, Houston, TX 77030, USA; E-Mails: uddalakb@bcm.edu (U.B.); mmk@bcm.edu (M.M.K.); eckols@bcm.edu (T.K.E.); mikhail02.22@gmail.com (M.K.); Pzl2@cornell.edu (P.L.); 2 Department of Molecular & Cellular Biology, Baylor College of Medicine, Houston, TX 77030, USA; E-Mails: krchrist@bcm.edu (K.C.); deane@bcm.edu (D.P.E.); 3 Department of Pathology & Immunology, Baylor College of Medicine, Houston, TX 77030, USA; 4 Department of Biochemistry & Molecular Biology, BCM 286, Room N-1319, Baylor College of Medicine, Houston, TX 77030, USA

**Keywords:** Stat3β, Stat3 beta, isoform, alternative RNA splicing, CT7, phosphorylation, monoclonal, oncogenesis, breast cancer, regulation

## Abstract

Since its discovery in mice and humans 19 years ago, the contribution of alternatively spliced Stat3, Stat3β, to the overall functions of Stat3 has been controversial. Tyrosine-phosphorylated (p) Stat3β homodimers are more stable, bind DNA more avidly, are less susceptible to dephosphorylation, and exhibit distinct intracellular dynamics, most notably markedly prolonged nuclear retention, compared to pStat3α homodimers. Overexpression of one or the other isoform in cell lines demonstrated that Stat3β acted as a dominant-negative of Stat3α in transformation assays; however, studies with mouse strains deficient in one or the other isoform indicated distinct contributions of Stat3 isoforms to inflammation. Current immunological reagents cannot differentiate Stat3β proteins derived from alternative splicing *vs.* proteolytic cleavage of Stat3α. We developed monoclonal antibodies that recognize the 7 C-terminal amino acids unique to Stat3β (CT7) and do not cross-react with Stat3α. Immunoblotting studies revealed that levels of Stat3β protein, but not Stat3α, in breast cancer cell lines positively correlated with overall pStat3 levels, suggesting that Stat3β may contribute to constitutive Stat3 activation in this tumor system. The ability to unambiguously discriminate splice alternative Stat3β from proteolytic Stat3β and Stat3α will provide new insights into the contribution of Stat3β *vs.* Stat3α to oncogenesis, as well as other biological and pathological processes.

## 1. Introduction

Signal transducer and activator of transcription (Stat) 3 belongs to a family of seven proteins (STATs 1, 2, 3, 4, 5A, 5B and 6) involved in signal transduction downstream of cytokine and growth factor receptors. Stat3 activation involves its phosphorylation on Y705 by Jak or receptor intrinsic tyrosine kinases followed by it homodimerization and accumulation within the nucleus [1]. Stat3 plays important roles in acute phase (stress) responses [2,3,4]. Constitutively tyrosine-phosphorylated (p) Stat3 has been found and implicated in the initiation and progression of many cancers, including breast [5], pancreas [6,7,8], head and neck squamous cell carcinoma (HNSCC) [9], leukemias, and lymphomas [10,11].

**Figure 1:**
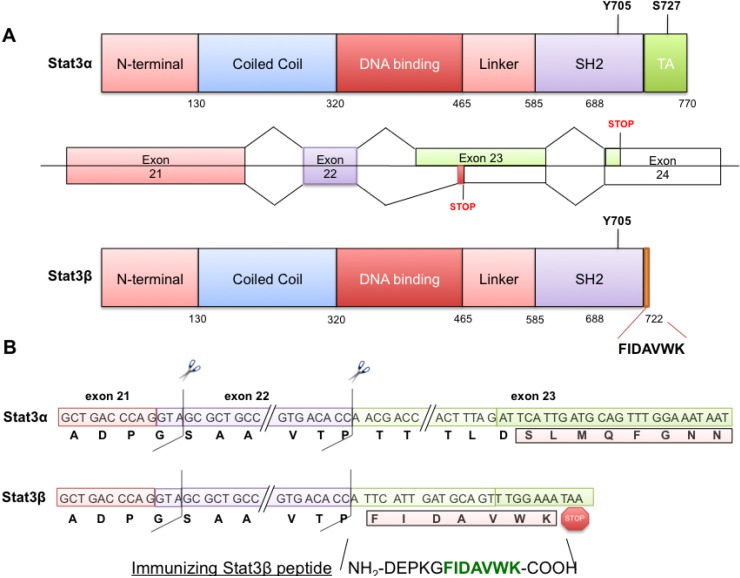
Schematic overview of domains of Stat3α and Stat3β and their derivation by normal splicing of the Stat3 gene or alternative splicing, respectively. Use of an alternative 3' splice acceptor site in exon 23 of the Stat3 gene (**A**, middle panel) generates the Stat3β isoform (**A**, lower panel) with the loss of 50 nucleotides from the exon and a frame shift, to add 21 nucleotides coding seven unique amino acids followed by a stop codon, within the exon 23 (**A**, **B** lower panels). This leads to a truncated Stat3β in place of the normally intron-spliced Stat3α (**A**, **B** upper panels).

Like most other STAT proteins, two major isoforms of Stat3 are found in cells; the more abundant full length (770 aa, 92 kDa) Stat3α and the less abundant truncated (722 aa, 83 kDa) Stat3β, generated through alternate splicing of exon 23 [12]. Stat3β lacks the 55 amino acid, C-terminal, trans-activation domain and due to a change in reading frame, also has an insertion of 7 unique amino acids (the CT7 epitope) at its carboxy terminal end [12] (Figure 1). Another truncated form of Stat3 has been identified that is similar in size to Stat3β (approximately 83 kDa) but is generated by protease-mediated cleavage of Stat3α [13]. This form of Stat3β (referred to as Stat3β-deg) lacks the unique CT7 epitope.

The ratio of Stat3α to Stat3β in various cells, ranges from 4:1 to 10:1 at the mRNA level, and 1:3 to 10:1 at the protein level [12,14,15], which suggested that the functions of the two isoforms may be non-overlapping [12]. Because Stat3β lacks the transactivation domain, it was initially thought to be ineffective in activating gene transcription and that it acted as a dominant negative reducing Stat3α transcriptional function [16]. However other studies clearly revealed a role of Stat3β in gene transcription [17,18] through cooperation with c-Jun [19,20] or through forming hetero dimers with Stat3α [21]. Mice selectively lacking Stat3β were found to be viable and fertile compared to mice lacking both Stat3 isoforms; macrophages from Stat3β-deficient mice demonstrated normal production of IL-6, IL-1β and TNF-α following LPS-treatment [22]. However, these mice were hypersensitive to endotoxin-induced inflammation and were able to both upregulate and downregulate multiple genes, indicating distinct transcriptional functions [22]. Comparing mice that specifically lacked either Stat3α or Stat3β, Maritano et al also confirmed that in contrast to Stat3β, which is not required for viability, Stat3α-deficient mice died within 24 h of birth [23]; however, Stat3β could rescue the embryonic lethality at 6.5 days of a total Stat3 deletion. Their findings also clearly indicated a role for Stat3β in inflammation and indicated that Stat3β, rather than being simply a dominant negative of Stat3α can activate a set of specific Stat3 target genes [23]. Stat3β also, was reported to play a major role in G-CSF-induced granulocytic differentiation of normal CD34+ myeloid progenitors [14,15,24,25]; activation and/or overexpression of Stat3α during this process resulted in proliferation rather than differentiation [26].

Cellular characterization of Stat3 isoforms revealed that Stat3β translocates to the nucleus either at the same rate [27] as Stat3α or a little faster [28]; once translocated, it is retained in the nucleus for much longer than Stat3α [27,28]. In addition, EGF-activated Stat3β is more stable and binds target DNA with higher affinity than equimolar concentrations of EGF-activated Stat3α [20] presumably, because of the dimer-destabilizing effect of the acidic C-terminal transactivation domain of Stat3α [20] absent from Stat3β. Stat3β also showed constitutive DNA-binding activity in the absence of EGF stimulation indicating a propensity of the isoform to form active, stable dimers capable of increased and stable DNA-binding [20]. Overexpression of Stat3β, but not Stat3α, along with the erythropoietin receptor-gp130 chimeric receptor in Cos-7 cells, led to nuclear translocation and increased DNA-binding activity even in absence of erythropoietin stimulation [29]. Transfection of a mutated Stat3 (Δ715, similar to natural Stat3β) also demonstrated constitutive activity suggesting the loss of negative regulation of both truncated proteins as a likely cause of ligand-independent (constitutive) activation. Recent studies using inducible expression of either Stat3α or Stat3β in Stat3^−/−^(null) murine embryonic fibroblasts (MEFs) also confirmed the prolonged nuclear translocation as well as prolonged phosphorylation of Stat3β by oncostatin M (OSM) compared to Stat3α [28]. Furthermore, co-expression of Stat3β also increased and prolonged phosphorylation of Stat3α [28]. We hypothesized, based on these recent observations that increased expression of Stat3β might contribute to the increased constitutive levels of pStat3 frequently observed in cancer cells.

Current immunological reagents are not specific for Stat3β, in particular, they are unable to differentiate Stat3β protein derived from alternative splicing from Stat3β protein resulting from proteolytic cleavage of Stat3α [13]. To test our hypothesis and to efficiently explore issues associated only with the Stat3β isoform formed by alternative splicing, we developed monoclonal antibodies that specifically recognize the 7 C-terminal amino acids (CT7 epitope, Figure 1) unique to Stat3β and that do not cross-react with Stat3α. We confirmed their stringent specificity using protein extracts from cells overexpressing either isoform, as well as purified recombinant Stat3 proteins with or without the CT7 epitope amino acids. We also used these antibodies to examine Stat3 isoform expression in a variety of cells, in particular, to assess the contribution of Stat3β to overall pStat3 levels within normal and cancer cells. Our results demonstrate that Stat3β levels positively correlate with overall pStat3 levels, a finding at odds with its previously described dominant-negative anti-oncogenic role [16,30,31]. These studies indicate that the ability to unambiguously discriminate alternatively spliced Stat3β from Stat3β derived from proteolytic cleavage of Stat3 and from full-length Stat3α will provide new insights into the roles of Stat3β *vs.* Stat3 in oncogenesis.

## 2. Results

### 2.1. Stat3β Immunogen Design and Mouse Immunization

Stat3 exists primarily as two isoforms, the longer form Stat3α (770 aa, 92 kDa) and the truncated Stat3β (722 aa, 83 kDa), which are expressed at the protein levels at approximately the ratio 4:1 (range from 4:1 to 10:1 at the mRNA level and from 1:3 to 10:1 at the protein level) in various cells [12,14,15]. Stat3α, the predominant splice form, is generated through splicing involving strong 5' splice donor sites, branch points, poly-pyrimidine tracts and 3' splice acceptor sites, present within intronic sequences. The Stat3β spliced form is generated by the use of an alternate, weaker splice acceptor site (as well as branch point and polypyrimidine tract) situated within the exon 23, leading to an altered reading frame and creating the addition of a stretch of seven unique amino acids (FIDAVWK/Phe-Ile-Asp-Ala-Val-Trp-Lys, Figure 1) followed by the introduction of a stop codon, thereby eliminating 55 amino acids from the C-terminal end of full length Stat3α. We added an additional 5 amino acids to this Stat3β-unique sequence and designed our immunizing peptide with the sequence DEPKGFIDAVWK (Asp-Glu-Pro-Lys-Gly-Phe-Ile-Asp-Ala-Val-Trp-Lys). Five mice, numbered 146–150, were immunized a total of four times (first immunization followed by first, second and third booster immunizations) with two weeks between immunizations. Mice were bled 13 days after the second and third boosts. We checked the antibody titer of the mice by ELISA after the second and third boosts.

### 2.2. Antisera from Mice Immunized with CT7 Peptide Specifically Detect Stat3β by Immunoblotting

Antisera from the five mice were tested for reactivity against Stat3β peptide, using ELISA (Supplemental Figure S1, Supplemental Table S1). Either the free peptide or BSA-conjugated peptide was immobilized on the plate to measure antibody titer in serum derived from immunized mice. Compared to the PBS-Free (or NMS-Free) and 1% BSA-PBS (or 1% BSA-NMS) controls, sera from all five immunized mice showed significant reactivity to both the free peptide or BSA-conjugated peptide, although BSA-bound peptide was generally more efficient for antibody capture. We then tested these anti-sera for their ability to detect specifically Stat3β by immunoblotting. Whole protein from 293 T cells mock transfected (transfection reagent only) or transiently (48 h) transfected with plasmids encoding either GFP-Stat3β, or GFP-Stat3α were separated by SDS-PAGE and transferred to nitrocellulose membranes and probed with the anti-sera from 5 mice as well with a monoclonal antibody (MoAb) against total Stat3 (tStat3; clone 124H6, Cell Signaling Technology). The tStat3 MoAb could detect (Supplemental Figure S2) both GFP-Stat3β (approximately108 kDa) and GFP-Stat3α (approximately117 kDa). Antisera from mouse #147 and #148 clearly could detect only the GFP-Stat3β, without any detection of GFP-Stat3α (Supplemental Figure S2).

### 2.3. Generation and Subcloning of Hybridomas

We chose mouse #147 (Supplemental Figure S2) to generate hybridoma clones by fusing the isolated splenocytes with immortalized myeloma cells. Fifteen 96-well plates of hybridomas were generated from this fusion and screened for reactivity against Stat3β peptide by ELISA. Positives were selected based on an ELISA OD that was greater than 0.3, most positives having ODs that were greater than 1.5 [32]. There were 29 positives chosen after the first ELISA. Eight out of 29 were chosen based on their lack of substantial reactivity to an unrelated peptide (ADRP) in a second ELISA and followed over time in culture and screened again a third time. Three positive clones (516, 954 and 1488) were selected, based on the presence of activity to both free peptide as well as BSA-conjugated peptide, and lack of reactivity against the non-related peptide. These and three additional clones 364, 1314 and 1412 were expanded and simultaneously screened by ELISA for ability to detect GFP-Stat3β by immunoblotting (Supplementary Figure S3). Culture supernatants from both 516 and 1488 could specifically detect GFP-Stat3β, with no reactivity to GFP-Stat3α. Although the 954 sup did not detect any band, we still selected all three clones 516, 954 and 1488 for sub-cloning. Sub-clones from three clones were generated using limiting dilution and after ELISA screening, four high-titer sub-clones [32] were selected each from the three clones. Four sub-clones from 516 (516G4, 516G10, 516H2, 516H5, Supplementary Figure S4A), one sub-clone from 954 (954E9, Supplementary Figure S4B) and two sub-clones from 1488 (1488 D5 and 1488 G6) were all found to specifically detect GFP-Stat3β, without detecting GFP-Stat3α. Three sub-clones, each from 516 G10, 954 E9 and 1488 G6, were positive by ELISA. Out of these, one sub-clone from 516 G10 (516 G10H9), two sub-clones from 954 E9 (954 E9ED5, 954 E9E7) and one clone from 1488 G6 (1488 G6G5), all were tested by immunoblotting and found to specifically detect Stat3β and not Stat3α (Supplemental Figure S5).

### 2.4. IgG Purification

**Figure 2:**
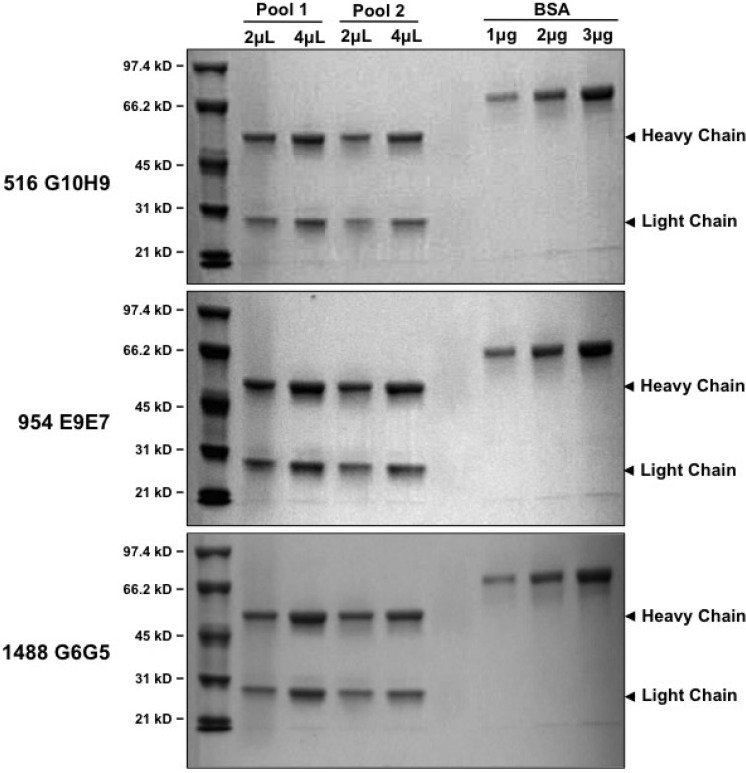
Coomassie stain of the purified Stat3β specific monoclonal antibodies. Ammonium-sulphate precipitated proteins were passed through protein G columns. The protein G-adsorbed antibodies were eluted and pooled together (pool 1). The flow through was again passed through the column to recover all unadsorbed antibodies and were similarly eluted and pooled (pool 2). Exactly 2 μL and 4 μL of pool 1 (lanes 2 and 3) and 2 μL and 4 μL of pool 2 (lanes 4 and 5) of pure antibodies were boiled with SDS-containing loading dye and ran on PAGE along with molecular weight markers (lane 1) and increasing amounts (1 µg, 2 µg, 3 µg) of control protein Bovine Serum Albumin (BSA, lanes 7–9) and stained with coomassie. Representative gels run as described above, for the three antibodies 516 G10H9 (upper panel), 954 E9E7 (middle panel) and 1488 G6G5 are shown.

IgG was purified from the bulk cultures of three clones, 516 G10H9, 954 E9E7 and 1488 G6G5 by ammonium sulphate precipitation and subsequent protein G purification. The protein G-adsorbed antibodies were eluted and pooled together (pool 1). The flow through was again passed through the column to recover all unadsorbed antibodies and were similarly eluted and pooled (pool 2). Purified antibodies (2 μL and 4 μL of pool 1, lanes 2 and 3; 2 μL and 4 μL of pool 2, lanes 4 and 5) were boiled with SDS-containing loading dye and separated by SDS-PAGE along with molecular weight markers (lane 1) and increasing amounts (1 µg, 2 µg, and 3 µg) of control protein BSA (lanes 7–9). Representative gels run as described above, for the three antibodies 516 G10H9 (upper panel), 954 E9E7 (middle panel) and 1488 G6G5 clearly show (Figure 2) the heavy and light chains in all the three monoclonals.

**Figure 3:**
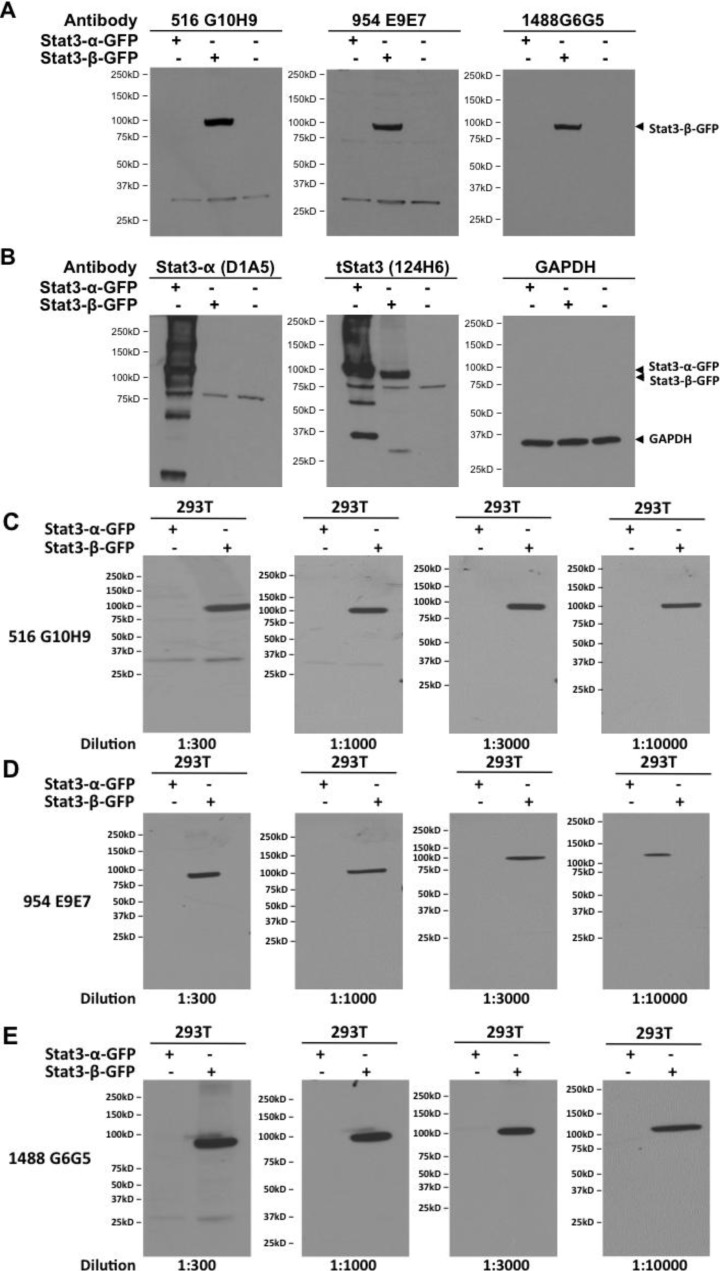
Stat3β monoclonals specifically detect Stat3β protein in cell protein extracts. Plasmids encoding fusion proteins Stat3α-GFP and Stat3β-GFP were transiently transfected into HEK 293T cells. Extracts (100 µg protein) from mock or plasmid transfected (48 h) cells were separated by SDS-PAGE, transferred to nitrocellulose membrane and replicate blots probed for (**A**) Stat3β using the three monoclonal antibodies 516 G10H9, 954 E9E7 and 1488 G6G5 and (**B**) Stat3α (Clone D1A5), total Stat3 (clone 124H6) and GAPDH and visualized by chemiluminiscence. Various dilutions (ranging from 1:300 to 1:10,000) of the three Stat3β monoclonal antibodies 516 G10H9 (**C**), 954 E9E7 (**D**) and 1488 G6G5 (**E**) were used to probe 100 µg protein from 293T cells transiently transfected with plasmids encoding fusion proteins Stat3α-GFP and Stat3β-GFP and visualized by chemiluminiscence.

The two pools of each antibody were pooled together and the protein quantity estimated to be approximately 1 mg/mL. These monoclonal antibodies were then tested (1:500 dilution) by immunoblotting and were found to detect specifically, GFP-Stat3β and not GFP-Stat3α (Figure 3A) transiently over-expressed in 293T cells, whereas the commercial tStat3 MoAb (clone 124H6) detected both, and the Stat3α-specific commercial MoAb (D1A5) specifically detected only GFP-Stat3α and not GFP-Stat3β (Figure 3B). Next, we checked the ability of various dilutions of the three monoclonals to specifically detect the same GFP-Stat3β overexpressing lysates (Figure 2C–E). Dilutions as low as 1:10,000 could clearly detect GFP-Stat3β specifically. Even at the highest concentration tested (1:300), none of the Stat3β-specific monoclonals detected Stat3α.

### 2.5. Dependence of Stat3β-Specific Monoclonal Antibodies to Detect Recombinant Stat3β upon Presence of the CT7 Epitope

**Figure 4:**
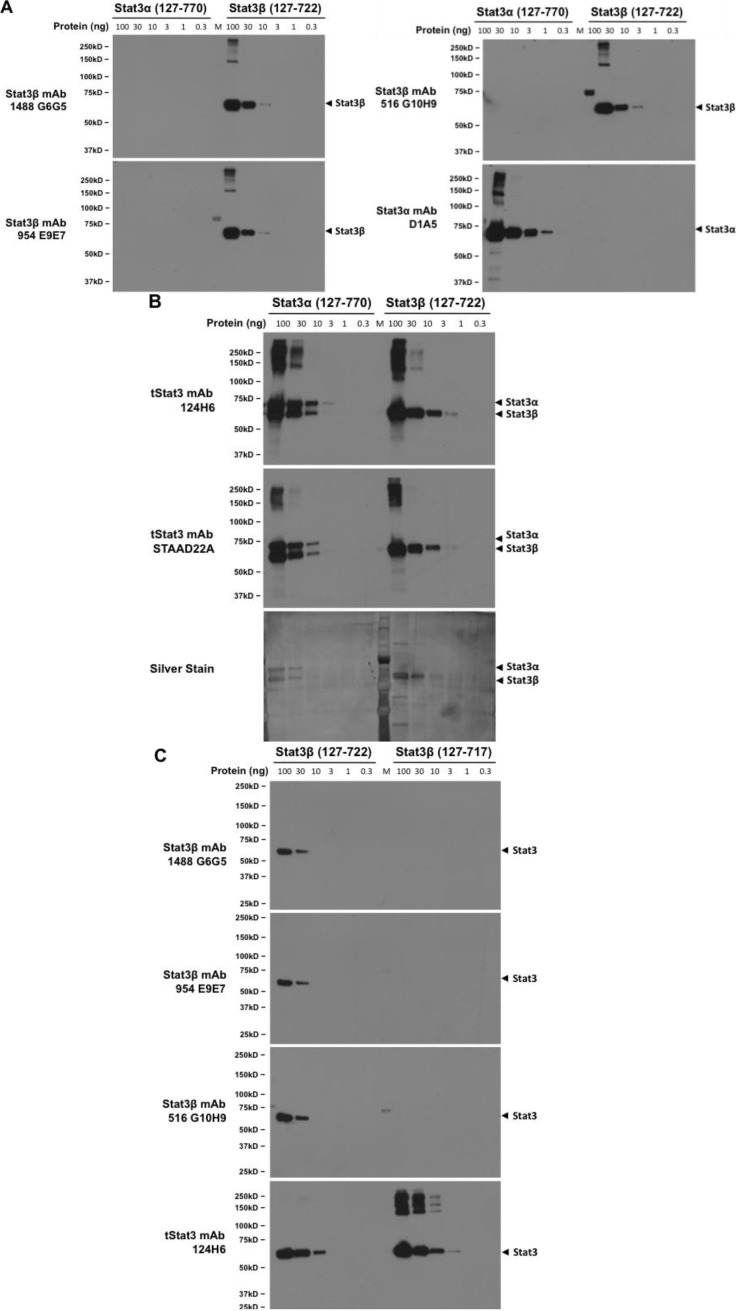
Stat3β monoclonal antibodies are specific for the CT7 epitope found exclusively on Stat3β. Truncated pure Stat3α (amino acids 127–770), truncated Stat3β (amino acids 127–722) and truncated Stat3β devoid of CT7 (amino acids 127–717) were purified from bacterial supernatants as described in the Methods section. Decreasing amounts (100 ng, 30 ng, 10 ng, 3 ng, 1 ng, 0.3 ng) of truncated Stat3α (amino acids 127–770), truncated Stat3β (amino acids 127–722) were loaded onto consecutive wells and subjected to SDS-PAGE, transferred to nitrocellulose membranes and probed (**A**) with the three monoclonal antibodies 516 G10H9, 954 E9E7 and 1488 G6G5 as well as (**B**) Stat3α (Clone D1A5), two total Stat3 antibodies, one raised against a C-terminal peptide (clone 124H6) and one raised against a N-terminal peptide (aa 149–262, clone STAAD22A) and visualized by chemiluminiscence. Protein in a replica gel was also detected using silver staining and shown at the bottom of panel (**B**). In panel (**C**), decreasing amounts (100 ng, 30 ng, 10 ng, 3 ng, 1 ng, 0.3 ng) of truncated Stat3β (amino acids 127–722) and truncated Stat3β devoid of CT7 (amino acids 127–717) were loaded onto consecutive wells and subjected to SDS-PAGE, transferred to nitrocellulose membranes and probed with the three monoclonal antibodies 516 G10H9, 954 E9E7 and 1488 G6G5 as well as total Stat3 antibody (clone 124H6) and visualized by chemiluminiscence.

Having demonstrated the specificity of the Stat3β monoclonals in lysates of cells overexpressing one or the other Stat3 isoform, we next wanted to further confirm that their specificity mapped to the 7 unique amino acids at the C-terminal of Stat3β. To this end, we cloned, expressed in bacteria, and purified three proteins—A truncated Stat3α devoid of 126 amino acids at its N-terminal end (Stat3α 127–770), a truncated Stat3β devoid of 126 amino acids at its N-terminal end, but with the CT7 epitope intact (Stat3β 127–722), and a truncated Stat3β devoid of 126 amino acids at its N-terminal end and also lacking the CT7 epitope (Stat3β 127–715). Decreasing amounts (100 ng, 30 ng, 10 ng, 3 ng, 1 ng, 0.3 ng) of the pure Stat3α 127–770 and the Stat3β 127–722 were boiled with gel loading dye and subjected to SDS-PAGE, transferred to nitrocellulose membrane and probed with the three Stat3β monoclonals and commercial antibodies able to detect only Stat3α (clone D1A5) or both Stat3α and β (clone 124H6 against C-terminal epitope, and clone STAAD22A raised against amino acid residues 149–662 of human Stat3). Each of the three Stat3β monoclonals detected only Stat3β 127–722 with the limit of detection being 10 ng at the 1:1000 dilution (Figure 4A). The Stat3α specific MoAb, on the other hand, detected only the Stat3α 127–770, although its sensitivity for Stat3α was higher (Figure 4A) than the Stat3β MoAbs with the limit of detection being 3 ng at 1:1000 dilution. In contrast, both the tStat3 MoAbs detected both Stat3α 127–770 and Stat3β 127–722 as shown in Figure 4B. Interestingly, along with the regular 73.1 kDa band corresponding to Stat3α 127–770, both tStat3 antibodies detected a lower molecular weight band roughly corresponding in size to Stat3β 127–722 (approximately 68.2 kDa). This band presumably represents degradation of Stat3α (127–770). As expected, the three Stat3β monoclonals did not detect this molecular weight-matched degraded Stat3β (Stat3β-deg), but could only detect Stat3β with the CT7 epitope, representing the alternative spliced form (Stat3β-spl). To further confirm the CT7 specificity of the Stat3β monoclonals, decreasing amounts of the Stat3β (127–722) and the truncated Stat3β missing the CT7 amino acids, Stat3β 127–715, were subjected to SDS-PAGE and immunoblotted with the Stat3β monoclonals and the tStat3 MoAb (clone 124H6). As Figure 4C shows, each Stat3β monoclonal detected Stat3β (127–722), which contains the CT7 amino acids, but not truncated Stat3β 127–717, which is missing the CT7 amino acids. These findings convincingly establish that each recognizes only the CT7 epitope unique to Stat3β. Thus, they represent the ideal tool for identifying Stat3β-spl as opposed to Stat3β-deg. We increased the exposure time from 1' (Figure 4C) to 2' (Supplemental Figure S6) and still could not demonstrate any Stat3β (127–717) being detected by the monoclonals, further indicating their high degree of specificity. The high sensitivity of the antibodies to detect Stat3β (127–722) is apparent from a 10 sec exposure shown in (Supplemental Figure S6).

### 2.6. Stat3β Monoclonal Antibodies Immunoprecipitate Stat3β from Cell Line Lysates

**Figure 5:**
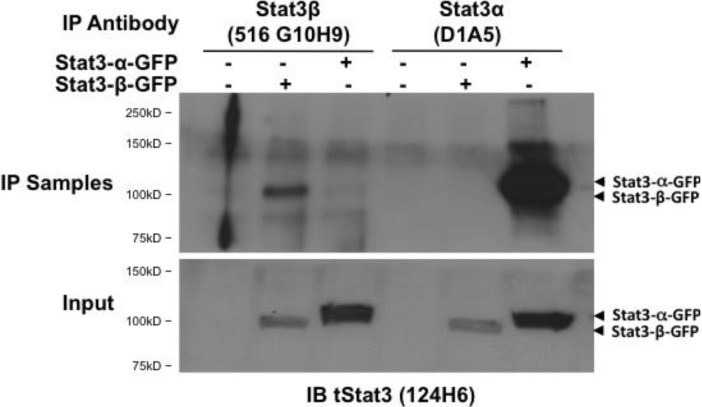
Stat3β monoclonal antibodies specifically immunoprecipitate Stat3β from protein lysates. Stat3β Mab 516G10H9 (1:30) and Stat3α Ab (D1A5) were added to 200 µg of protein from either untransfected Stat3^−/−^murine embryonic fibroblasts (MEFs) or cells transiently transfected with plasmids encoding fusion proteins Stat3α-GFP and Stat3β-GFP, incubated overnight at 4 °C. The lysates were then incubated with protein G-agarose beads for 30 min at 4 °C and after centrifugation, the beads boiled in loading dye and the eluted proteins subjected to SDS-PAGE, transferred to nitrocellulose membrane and probed for total Stat3 (clone 124H6, upper panel). An equivalent volume of cell lysates (20 µL) used in each reaction were subjected to SDS-PAGE, transferred to nitrocellulose membrane and probed for total Stat3 (clone 124H6, lower panel) to indicate the amount of protein input.

GFP-Stat3α or GFP-Stat3β were over expressed in Stat3^−/−^MEFs and protein lysates (500 µg) from each were immunoprecipitated using Stat3β MoAb 516 G10H9 (left 3 wells) or Stat3α MoAb, D1A5 (right three wells) and probed with tStat3 MoAb (clone 124H6) that detects both Stat3α and Stat3β. The results clearly show that the Stat3β could pull down only GFP-Stat3β and not GFP-Stat3α (Figure 5). Similar results were obtained using the other two antibodies 1488 G6G5 and 516 G10H9 [33].

### 2.7. Stat3β Monoclonal Antibodies Detect Endogenous Stat3β in Cell Line Lysates Revealing that Stat3β Levels Correlated to pStat3 Levels in Breast Cancer Cell Lines

**Figure 6:**
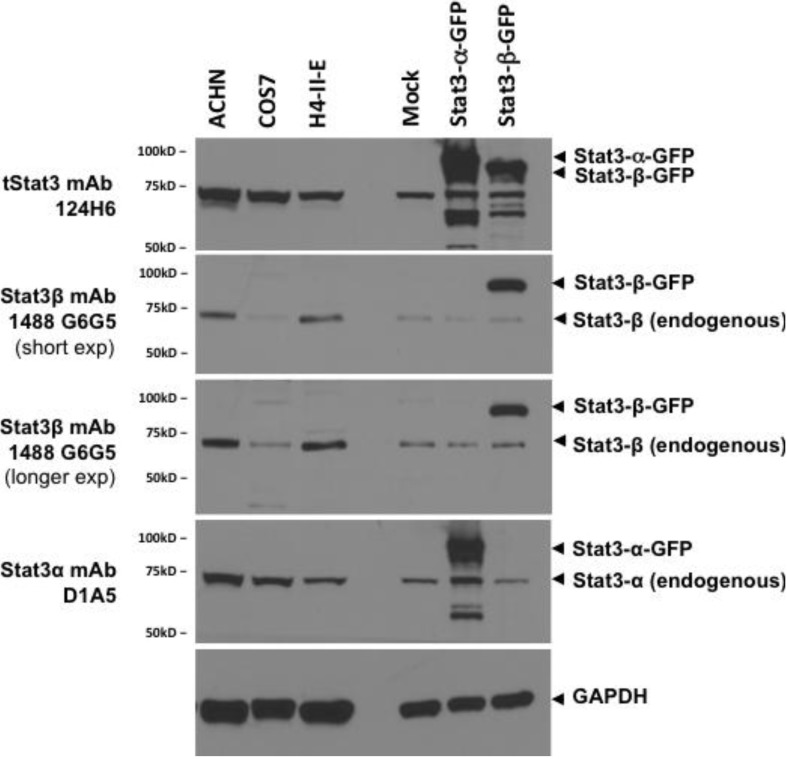
Stat3β monoclonal antibody can detect endogenous Stat3β in lysates from cell lines expressing various levels of Stat3β. Lysates (100 µg of total protein) from cell lines ACHN, COS7, and H4IIE (lanes 1–3) were separated by SDS-PAGE, transferred to nitrocellulose membrane and replicate blots probed for total Stat3 (clone 124H6), Stat3β (516G10H9), Stat3α (Clone D1A5) and GAPDH and visualized by chemiluminiscence. Lysates (30 µg of protein) from 293T cells, mock transfected (transfection reagent alone) or transiently transfected with plasmids encoding fusion proteins Stat3α-GFP and Stat3β-GFP were used as controls (lanes 5–7).

Protein extracts (100 µg) from semi-confluent cultures of three cell lines known to express increased amounts of Stat3β, were immunoblotted with Stat3β (clone 1488 G6G5), tStat3 (clone 124H6), and Stat3α (clone D1A5) and GAPDH. Protein extracts (30 µg) from 293T cells mock transfected or overexpressing GFP-Stat3α or GFP-Stat3β were used as controls. MoAb 1488 G6G5 detected endogenous Stat3β in all cells under conditions when GFP-Stat3β was expressed (Figure 6).

**Figure 7:**
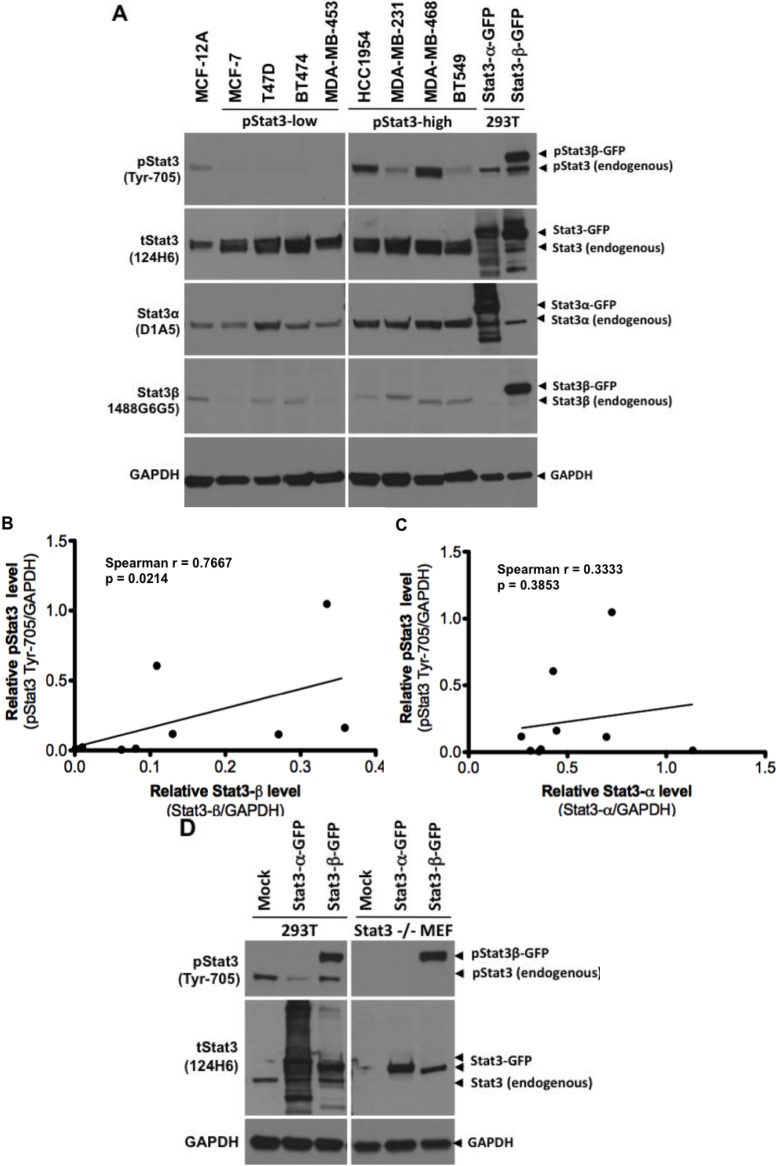
Stat3β overexpression correlates with Stat3 phosphorylation in breast cancer cell lines. In panel (**A**), lysates (100 µg protein) from normal breast epithelial cells MCF-12A, and breast cancer cell lines MCF-7, T47D, BT-474, MDA-MB 453, HCC1954, MDA-MB-231, MDA-MB-468, and BT549 cells were subjected to SDS-PAGE, transferred to nitrocellulose membrane and probed for total pStat3 (Tyr-705, clone D3A7), tStat3 (clone 124H6), Stat3α (clone D1A5), Stat3β (1488 G6G5) and GAPDH. Lysates (30 µg of protein) from 293T cells, mock transfected (transfection reagent alone) or transiently transfected with plasmids encoding fusion proteins Stat3α-GFP and Stat3β-GFP were used as controls (lanes 10–11). Stat3β and GAPDH levels were quantified by densitometry and the GAPDH-normalized pStat3 levels plotted as a function of their corresponding Stat3β levels (**B**, Spearman r = 0.7667, *p* < 0.05) or Stat3α levels (**C**, Spearman r = 0.3333, *p* > 0.05). In panel **D**, Stat3β-GFP was constitutively phosphorylated when overexpressed in both 293T cells (left panel) and Stat3^−/−^MEFs (right panel), while Stat3α-GFP was not.

Whole cell lysates (100 µg) from eight breast cancer cell lines—Four with low constitutive pStat3 levels (MCF7, T47D, BT474 and MDA-MB-453 [5]) and four with increased constitutive pStat3 levels (HCC1954, MDA-MB-231, MDA-MB-468, and BT549)—Were subjected to SDS-PAGE, transferred to nitrocellulose membranes and probed for total pStat3 (Tyr-705, clone D3A7), tStat3 (clone 124H6), Stat3α (clone D1A5), Stat3β (1488 G6G5) and GAPDH (Figure 7A). Whole cell lysates (30 µg) of 293T cells mock transfected or overexpressing GFP-Stat3α or GFP-Stat3β were used as controls. Levels of pStat3, Stat3β and GAPDH were quantified by densitometry and the GAPDH-normalized pStat3 levels in the cell lines correlated to the corresponding Stat3β/GAPDH levels. Results indicated that the level of pStat3/GAPDH in 8 cell lines positively correlated with levels of Stat3β/GAPDH (Figure 7B; Spearman r = 0.7667, *p* = 0.0214). This, to our knowledge, is the first report of a correlation between Stat3β levels and levels of constitutive Stat3 phosphorylation in cancer cells.

Of note, the control lysates from 293T cells overexpressing Stat3β-GFP also demonstrated markedly increased phosphorylation (Figure 7A) while, the lysates from cells overexpressing Stat3α-GFP did not. Identical results were obtained when Stat3β-GFP was overexpressed in a second cell line, Stat3^−/−^MEFS [27,34] (Figure 7D). These findings suggest that increased expression of Stat3β alone in breast cancer cell lines may directly contribute to the increased constitutive levels of pStat3 observed.

## 3. Discussion

Since its discovery in mice and humans 19 years ago, the contribution of Stat3β, the alternatively spliced isoform of Stat3, to the overall functions of Stat3 has been controversial due, in part, to the unavailability of reagents capable of unambiguously distinguishing Stat3β from Stat3α, the more common isoform, and its degradation products within cells containing both isoforms [12,14,15,20,22,23,24,25,27,28,31]. Although initially thought to be transcriptionally inactive and even a dominant negative isoform, which opposes Stat3α transcriptional function [16,30], recent studies strongly support the concept that Stat3β has unique functions distinct from Stat3α, such as protection from endotoxic shock [22], modulating the response to inflammatory cytokines [23] and hemorrhagic shock [35,36,37,38], granulocyte differentiation [14,15,24,25], perhaps through its ability to affect transcription of multiple genes either alone, or in conjunction with Stat3α and/or c-jun [3,19,21,22,23,24,28]. To help resolve some of the controversies regarding its functions and explore new ones, we developed Stat3β specific mouse monoclonal antibodies, using a peptide encompassing the Stat3β-unique seven amino acids (CT7) as immunogen. We extensively tested these antibodies using protein lysates from cells overexpressing GFP-Stat3β or GFP-Stat3α, as well as using recombinant Stat3 proteins with or without the CT7 epitope. Our results show each monoclonal antibody specifically detects Stat3β and does not detect Stat3α. Furthermore, the ability of each monoclonal antibody to detect Stat3β protein depended on the protein containing the CT7 epitope. We used the antibodies, along with others purchased commercially, to examine the levels of Stat3β, Stat3α and pStat3 in a panel of breast cancer cell lines and found, for the first time for any cancer system, that pStat3 levels within breast cancer cells positively correlated with the level of Stat3β expression within these cell lines. Given the abundance of evidence correlating the presence of phosphorylated Stat3 (pStat3), especially pStat3 localized to the nucleus, to oncogenesis and aggressive tumor cell behavior, our findings suggest that increased Stat3β levels contribute to pStat3 levels, and hence oncogenesis and more aggressive behavior in breast cancer.

Immunoblotting of pancreatic cancer cell lines previously suggested there was increased Stat3β in those cell lines with increased constitutive pStat3 activation [39]. However, the identification of Stat3β in these studies was determined based upon band migration following SDS-PAGE and immunoblotting using antibody to total Stat3, and not the Stat3β-specific antibodies described here. This additional analysis currently is underway in our laboratory. The association between increased levels of Stat3β with increased constitutive pStat3 levels and, consequently oncogenesis, is in contrast to previously published findings demonstrating that Stat3β overexpression antagonized the transforming effects of Stat3α in NIH-3T3 transformation assays [23,24,40] as well as the more recent finding [31] that an oligonucleotide-mediated switching to preferential splicing to Stat3β (rather than Stat3α) promoted anti-tumorigenic behavior. However, the earlier studies [16,40] and the oligonucleotide switch studies were limited to a single cell line (NIH-3T3 or MDA-MB-435, respectively). The latter cell line [31] was originally thought to be a breast cancer cell line, but more recently has been authenticated to be a melanoma cell line [41] Overexpression of Stat3β in some tumor cells (melanoma, prostate, ovarian and breast cancer [42,43,44] led to growth suppression *in vitro* and *in vivo*, whereas in AML patient samples, increased Stat3β expression was associated with shorter overall patient survival [45,46]. Thus, the effect of Stat3β on oncogenesis and tumor cell behavior may depend on the cell type, as well as the level of overexpression. The availability of Stat3β monoclonal antibodies will allow a systematic examination of the association of Stat3β expression with pStat3 levels in multiple tumor systems, which will determine the breadth of the association between levels of Stat3β and pStat3 that we identified in breast cancer cells.

Our group and others demonstrated that Stat3β has increased nuclear retention upon stimulation by IL-6 [27] or OSM [28]. The latter group, using Stat3^−/−^MEFs engineered to inducibly express either Stat3 isoform, also showed that Stat3β exhibited increased and prolonged phosphorylation, along with more rapid nuclear translocation following OSM exposure [28]. In addition, the latter group revealed that co-expression of both isoforms revealed cross regulation, whereby Stat3β enhanced and prolonged Stat3α phosphorylation [28]. Similarly, another group demonstrated that adenoviral delivery of Stat3β followed by IL-6 stimulation, potentiated Stat3α phosphorylation [47]. One of the major pathways to increased pStat3 levels in breast cancer cells involves IL-6 [48]; thus, the studies summarized above provide a possible mechanism to explain our finding of increased pStat3 levels in breast cancers expressing increased levels of Stat3β. Prolonged phosphorylation of Stat3β has been ascribed to the reduced ability of phosphatases to bind to and/or dephosphorylate it compared to pStat3α [28]. Similarly, the heterodimer of pStat3α and pStat3β is more stable than a homodimer of pStat3α. Of note, mutation of Stat3β at R609L (Stat3βR609L), a residue within the SH2 domain critical for homodimerization, abrogated the effect of Stat3β overexpression on prolonging Stat3α phosphorylation [28].

The question arises as to the mechanism of the increase in Stat3β expression in breast cancer cells. It could relate to a general increase in the expression and function of the spliceosome machinery components in these cancer cells [49] or to alteration in a specific regulatory mechanism(s) normally controlling differential isoform generation. Recently miR146b has been reported to specifically degrade Stat3β in normal mammary luminal cells [50], suggesting this as a normal mode of regulation. Could the loss of this miRNA in breast cancer cells lead to an aberrant increase in Stat3β in these cells? Loss of miR146b gene (deletion of 10q24–26) has been reported in glioma [51], which supports this possibility. In fact, a miR46b-mediated negative feedback loop present in normal breast cell lines, which is activated by Stat3, is lost in breast cancer cell lines with sustained Stat3 activity [52,53]. In addition, levels of miR146b negatively correlated to nuclear pStat3 in 24 invasive breast tumor specimens [52,53]. Our antibodies likely will prove extremely useful in studies designed to examine the relationship between miR146b and Stat3β in cancer cells.

## 4. Experimental

### 4.1. Cell Lines

The human breast cancer cell lines (MCF-7, T47D, ZR-75-1, BT-474, MDA-MB 453, SKBR3, Hs578T, HCC1954, MDA-MB-231, MDA-MB-468, and Sum159PT), the breast epithelial cell lines (MCF10A and MCF-12A), and the AML cell line, Kasumi 1, were purchased from the American Type Culture Collection (ATCC, Rockville, MD, USA). Culture media, antibiotics, FBS, and other supplements were bought from Invitrogen (Grand Island, NY, USA). The rest were procured through the cell line core at Baylor College of Medicine. Cells were grown in their respective recommended complete growth media. MCF10A, MCF12A and Sum159PT cells were grown in DMEMF12 plus EGF/hydrocortisone. MCF-7, MDA-MB-453, Hs578T, MDA-MB-231, and MDA-MB-468 cells were grown in DMEM plus 10%FBS. T47D, ZR-75-1, BT-474, and HCC1954 cells were grown in RPMI-1640 plus 10% FBS. SKBR3 cells were grown in McCoy’s 5A plus 10% FBS. Cells were maintained in their respective complete media along with streptomycin, penicillin, amphotericin, Glutamax, and pyruvate and were not passed continuously more than 4 weeks. Cell lines were originally authenticated by ATCC, but no further authentication was done. The Stat3^−/−^murine embryonic fibroblast cell line (MEF) was a gift from Dr. Valeria Poli [27,54] and maintained in DMEM plus 10% FBS.

### 4.2. Stat3 Plasmids and Purification of Stat3 Protein

GFP-Stat3α and GFP-Stat3β were constructed as described before [27] from original Stat3α and Stat3β plasmids provided by Dr. Rolf Van de Groot [16]. The sequence encoding the Stat3α construct (amino acid 127–770), the Stat3β (amino acid 127–722) and the Stat3βΔCT7 construct (amino acid 127–717) were generated by PCR from original full length Stat3α or Stat3β clones [55] and cloned into pET15b (Novagen) expression vector and used to transform *Escherichia coli* strain BL21DE3. Protein expression was induced by adding 1 mM isopropyl-β-d-thiogalactopyranoside (IPTG) to the bacterial growth at an optical density of 0.6 at 600 nm. After incubation for 5 h at 21 °C, cells were harvested, resuspended in buffer containing 20 mM HEPES, pH 7.6, 0.2 M KCl, 20 mM DTT, 10 mM MnCl_2_ and 1 mM PSMF. The cells were lysed by sonication. Stat3 protein was isolated by precipitation with 35% ammonium sulfate; the resulting proteins were dialyzed in 20 Mm Tris, 5 mM DTT and purified by ion exchange chromatography using a Q-sepharose column. Purified Stat3 proteins were dialyzed in 20 mM HEPES, pH 7.6, 0.2 M NaCl, 5 mM DTT, 10 mM MgCl_2_ and stored at −80 °C until use.

### 4.3. Peptide Design and Mouse Immunization

Stat3α, the normal splice form is generated through splicing involving strong 5' splice donor sites, branch points, polymiridine tracts and 3' splice acceptor sites, present within intronic sequences (Figure 1). Stat3β spliced form is generated by the use of an alternate albeit weaker splice acceptor site (as well as branch point and polypyrimidine tract) situated within the exon 23, leading to an altered reading frame, creating the addition of seven unique amino acids (FIDAVWK/Phe-Ile-Asp-Ala-Val-Trp-Lys, Figure 1) followed by the introduction of a stop codon, thereby eliminating 55 amino acids from the C-terminal end of full length Stat3α. We added an additional 5 amino acids to the Stat3β-unique sequence and designed our immunizing peptide with the sequence DEPKGFIDAVWK (Asp-Glu-Pro-Lys-Gly-Phe-Ile-Asp-Ala-Val-Trp-Lys). Five mice, numbered 146–150, were immunized a total of four times, two weeks between immunizations. Mice were bled 13 days after the second and third boosts. We checked the antibody titer of the mice by ELISA after the second and third boosts.

### 4.4. Hybridoma Formation

Splenocytes from immunized mice were aseptically isolated and freed of fat, clots, and RBC, while serum separated from blood collected by cardiac puncture was stored at −80 °C. Myeloma cells were simultaneously prepared and then mixed with the splenocytes (1:4) and mixed thoroughly, media removed by centrifugation followed by addition of PEG1500/RPMI (50:50) in a dropwise manner over 1 min, with gentle stirring of the cell pellet while adding the PEG. Stirring was continued, while adding additional amounts of RPMI, over 1 to 3 min, so that the final cell suspension is free of cell clumps. Cells were then pelleted by centrifugation and resuspended in 10 mL of RPMI (with 15% FBS) to 3 × 10^6^ cells/mL and plated on 100 mm dishes and incubated overnight at 37 °C in 7.0% CO_2_. The next day, cells were harvested, centrifuged, and cell pellets resuspended in a RPMI (15% FBS) plus AHAT medium plus 1× Hybridoma Cloning Supplement in a 250 mL conical and mixed. 2 drops/well of this cell suspension was plated in fifteen 96 well CoStar tissue culture dishes, leaving the first column empty for controls at screening time. In plate 15, 1 row of the myeloma cells in AHAT was plated as a control. Plates were incubated at 37 °C plus 7.0% CO_2_ with replenishment by 2 drops/well of fresh AHAT medium and periodically, half of the fluid changed and replaced with 2 drops of AHAT medium. Supernatants were screened from hybridoma colonies when they were large enough to be producing good quantities of antibody (*i.e.*, 1/3 confluency, generally 10–12 days post-fusion). After the first screening, hybridomas were fed with RPMI plus 15% FBS plus AHT medium. After the two week period, the hybridomas were fed with RPMI plus 15% FBS alone.

### 4.5. ELISA for Detecting Anti-Stat3β Activity in Serum and Culture Supernatants

Seven rows of a 96 well Thermo Immulon 2 ELISA plate were coated with 50 µL/well of a 50 µg/mL concentration of Stat3β-BSA peptide. Seven rows of a second 96 well were coated with 50 µL/well of a 50.0 µg/mL concentration of Stat3β free peptide for O/N. The plate was washed, blocked with 200 μL/well 1% BSA/PBS 3 h at RT, washed and appropriate control and test antisera or supernatant samples were added and incubated overnight at 4 °C. The plates were then washed, secondary goat anti-mouse IgG (Fc specific)-HRP added, (1:5000 in 1% BSA/PBS) to each well and incubated, covered, for 2.5 h at RT. After another round of wash, the plates were developed, using 100 μL/well OPD as the substrate, and after 35 min, OD was read at 490 nM.

### 4.6. Purification of IgG from Culture Supernatants

IgG was precipitated from the 2 L cultures of each of the hybridomas (516 G10H9, 954 E9E10 and 1488 G6G5) by ammonium sulfate, centrifuged, supernatant removed, and protein pellets dissolved in 200 mL at 4 °C on a rocking platform, dialyzed overnight, centrifuged, and stored at 4 °C. Antibody was then adsorbed onto protein G column. The protein G-adsorbed antibodies were eluted and pooled together (pool 1). The flow through was again passed through the column to recover all unadsorbed antibodies and were similarly eluted and pooled (pool 2). The two pools, after the purity was verified by immunoblotting, were pooled to give the final antibody solution. The eluted fractions were treated with neutralization buffer (15.0 µL/1 mL) and immediately dialyzed in 1 L of PBS for 4–6 h, changing the buffer one time. The antibody solution were stored at −80 °C.

### 4.7. Immunoblotting

Whole cell protein extracts (100 µg) from normal breast epithelial cells (MCF-10A and MCF-12A), breast cancer cell lines (MCF-7, T47D, BT-474, MDA-MB 453, HCC1954, MDA-MB-231, MDA-MB-468, and BT549) and 293T cells either mock-transfected or transfected with GFP-Stat3α or GFP-Stat3β cells were subjected to SDS-PAGE, transferred to nitrocellulose membrane and immunoblotted for total pStat3 (Tyr-705, clone D3A7), tStat3 (clone 124H6), Stat3α (clone D1A5), Stat3β (1488 G6G5) and GAPDH. For Figure 7A, some cell lines with multiple and/or crooked bands (difficult to quantify pixel-density) were cut off and remaining portions juxtaposed. Because 100 µg of protein were loaded, two gels were used to accommodate all the cell lines and controls (run on each gel). After transfer to membrane, blocking, probing, *etc.*, the two membranes of the set (probed by a particular ab) were always exposed together on the same x-ray film for authentic comparison of the protein levels. Band densities obtained for pStat3, Stat3α, Stat3β and GAPDH were quantified by densitometry using the Image J software [56].

### 4.8. Immunoprecipitation

For immunoprecipitation of Stat3α and Stat3β proteins, Stat3^−/−^MEF cells were plated in 100 mm plates and transfected with GFP-Stat3 constructs using GeneJuice transfection reagent as per manufactures instructions (EMD Millipore). Forty-eight hours post transfection, the cells were washed in ice cold PBS and lysed in Triton Buffer containing 20 mM HEPES (pH 7.5), 150 mM NaCl, 1 mM EDTA, 1% Triton X-100, Roche protease and phosphatase inhibitor cocktails. Each lysate (100 µL) was diluted 5 times in HEPES buffer without Triton X-100. The diluted lysates were then incubated at 4 °C overnight with 3 µg each of antibodies to Stat3α (D1A5) or Stat3β (516 G10H9). The antibody/lysate mixtures were added to 60 µL equivalent of 50% aspirated magnetic protein A beads (Invitrogen) and incubated for 15 min on ice. The beads were washed three times with 500 µL of Wash buffer containing 20 mM HEPES (pH 7.5), 150 mM NaCl, 1 mM EDTA, 0.25% Triton X-100, Roche protease and phosphatase inhibitor cocktails and analyzed by SDS-PAGE and immunoblotted and probed for total Stat3 (clone 124H6). Each lysate (20 µL) was subjected to SDS-PAGE, transferred to nitrocellulose membrane and probed for total Stat3 (clone 124H6) to indicate the protein input.

## 5. Conclusions

We developed and characterized monoclonal antibodies recognizing the Stat3β-unique CT7 epitope that do not cross-react with Stat3α. Their use in immunoblotting studies revealed for the first time that levels of Stat3β protein in breast cancer cell lines positively correlated with overall pStat3 levels, suggesting that Stat3β may contribute to constitutive Stat3 activation in this tumor system. Use of these reagents to unambiguously discriminate splice alternative Stat3β from proteolytic Stat3β and Stat3α will provide new insights into the contribution of Stat3β *vs.* Stat3α to oncogenesis, as well as other biological and pathological processes.

## Supplementary Files

Supplementary File 1
