# Supplementary material for: Monoclonal Antibodies Specific for STAT3β Reveal Its Contribution to Constitutive STAT3 Phosphorylation in Breast Cancer
Source: Cancers (Basel). 2014 Sep 29;6(4):2012–34. doi: 10.3390/cancers6042012 (PMC4276954; doi:10.3390/cancers6042012)
Supplement: Supplementary File 1 [file cancers-06-02012-s001.pdf]

Supplement Materials

Figure S1. 96 well ELISA plates were coated with Stat3β-BSA peptide or Stat3β free peptide for O/N. The plate was washed, blocked with BSA in PBS, washed and appropriate control and test antisera from immunized mice #146–150 (**A**–**E**) were added and incubated overnight at 4 °C. The plates were then washed, incubated with secondary goat anti-mouse IgG (Fc specific)-HRP for 2.5 h. After another round of wash, the plates were developed, using OPD as substrate, and OD was read at 490 nM.

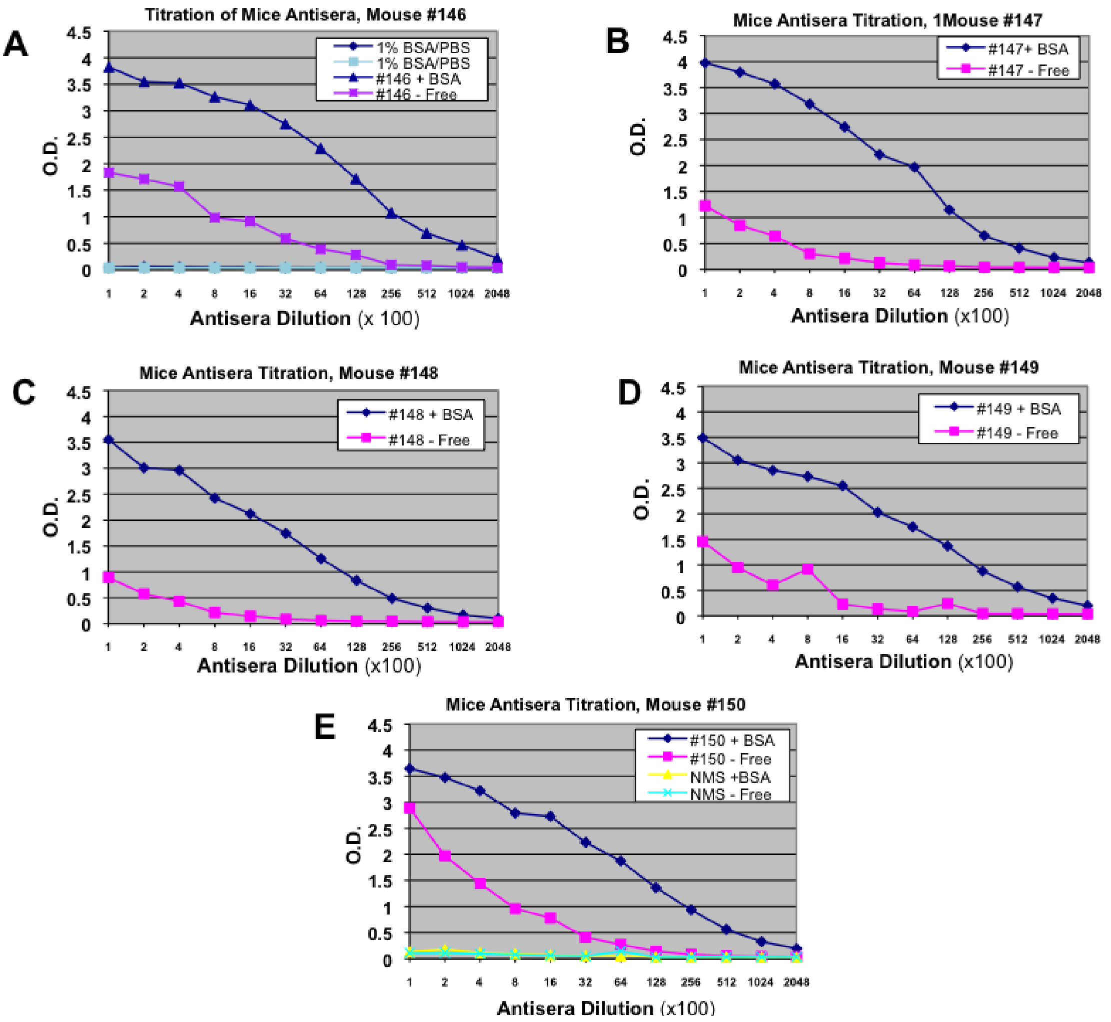

Figure S2. Screening of 6 hybridoma supernatants cloned from mouse #147 by immunoblotting, using lysates from 293 T cells transiently transfected with Stat3-α-GFP, Stat3-β-GFP.

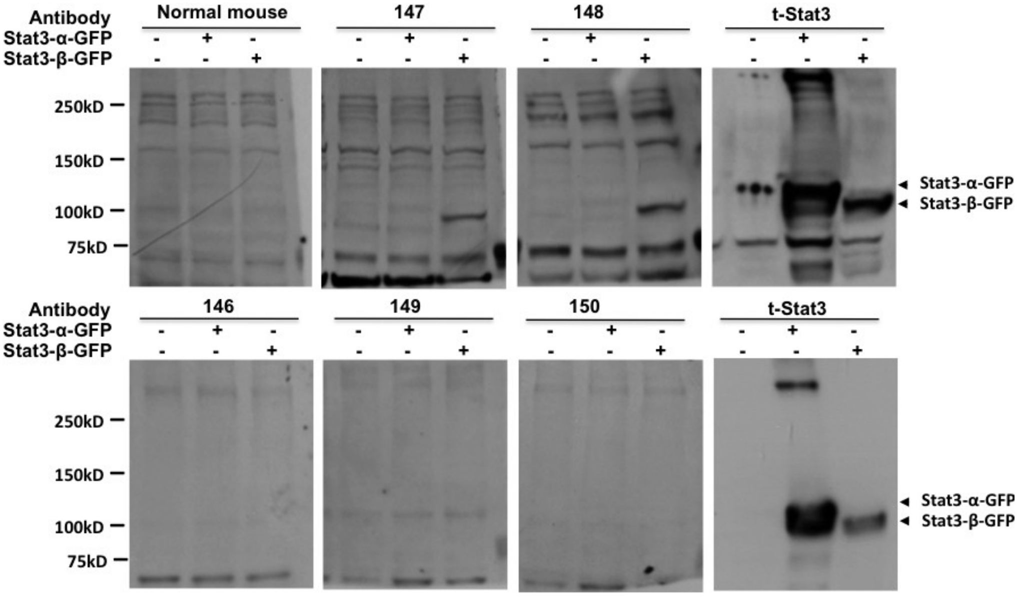

Figure S3. Screening of 6 hybridoma supernatants cloned from mouse #147 by immnoblotting using lysates from 293 T cells transiently transfected with Stat3-α-GFP, Stat3-β-GFP.

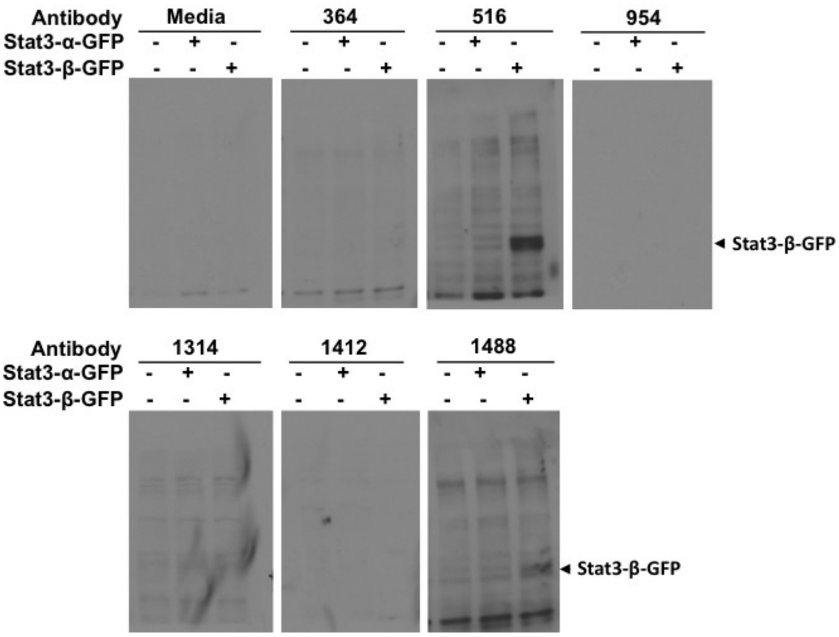

Figure S4. Screening of sups from sub clones of hybridoma clones (**A**) 516, and (**B**) 954, and 1488 by immunoblotting, using lysates from 293 T cells transiently transfected with Stat3-α-GFP, Stat3-β-GFP.

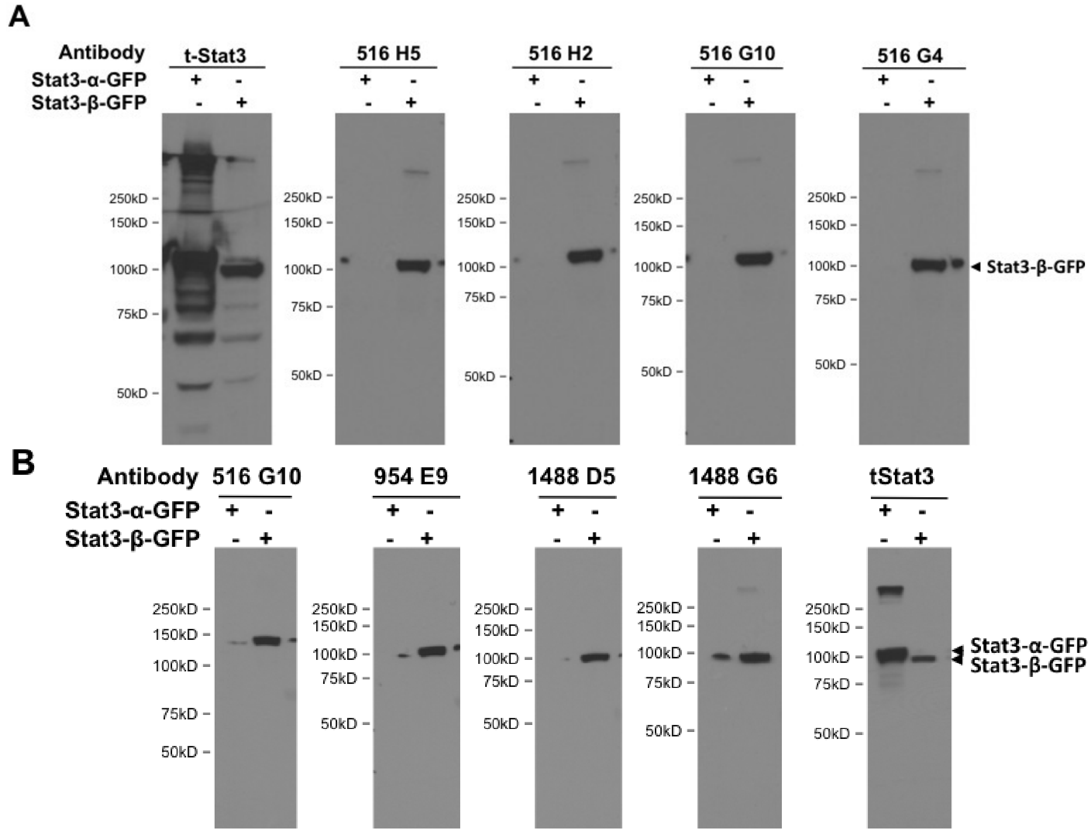

Figure S5. Screening of sups from 4 sub clones of hybridoma clones 516G10, 954E9, 1488G6 by immunoblotting, using lysates from 293 T cells transiently transfected with Stat3-α-GFP, Stat3-β-GFP.

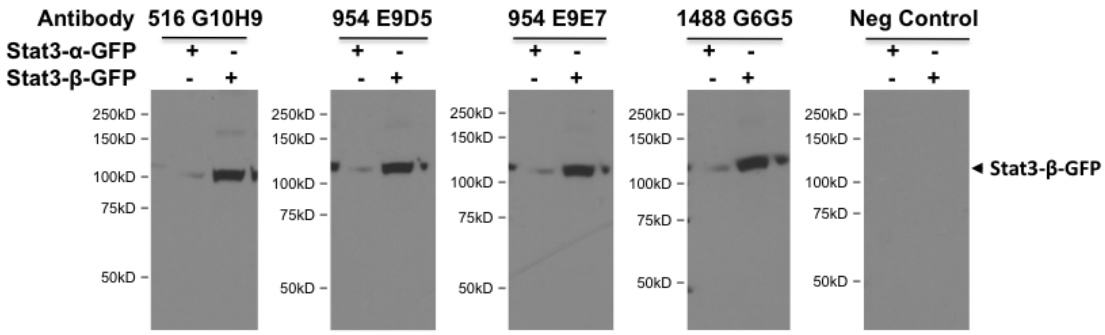

Figure S6. Decreasing amounts (100 ng, 30 ng, 10 ng, 3 ng, 1 ng, 0.3 ng) of truncated Stat3β (amino acids 127–722) and truncated Stat3β devoid of CT7 (amino acids 127–717) were loaded onto consecutive wells and subjected to SDS-PAGE, transferred to nitrocellulose membranes and probed with the three monoclonal antibodies 516 G10H9, 954 E9E7 and 1488 G6G5 as well as total Stat3 antibody (clone 124H6) and visualized by chemiluminiscence as described in Figure 4. The X-ray film was either exposed to the chemiluminiscence for either 10 s (**A**) or 2 min (**B**).

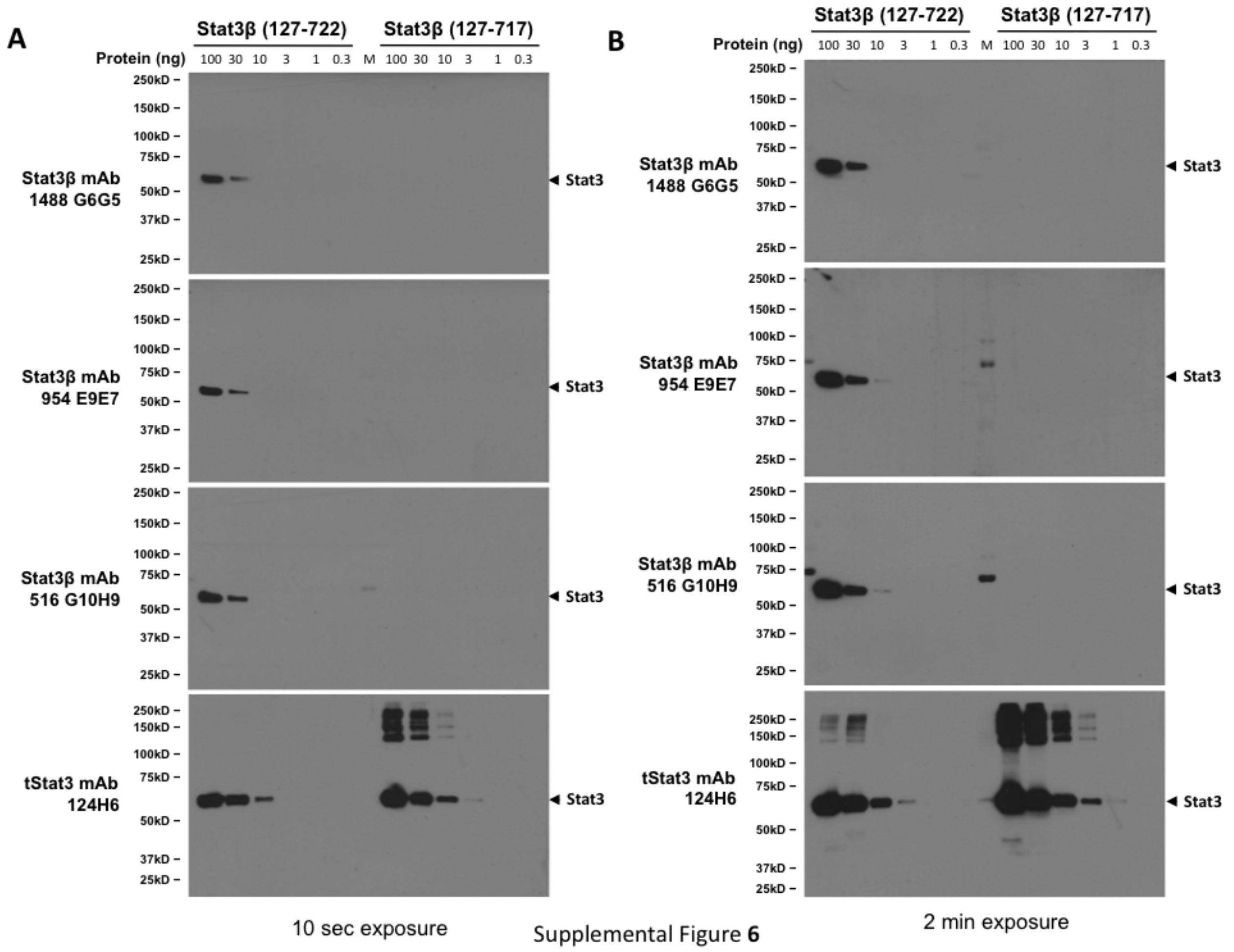

Table S1. Elisa data showing reactivity of anti-sera against Stat3-β immunizing peptide.

Dilution	1% BSA/PBS	1% BSA/PBS	#146 + BSA	#146 − Free	#147 + BSA	#147 − Free	#148 + BSA	#148 − Free	#149 + BSA	#149 − Free	#150 + BSA	#150 − Free	NMS + BSA	NMS − Free
100	0.048	0.037	3.824	1.833	3.976	1.228	3.552	0.889	3.492	1.46	3.645	2.889	0.132	0.114
200	0.063	0.038	3.544	1.711	3.798	0.85	3.006	0.578	3.056	0.947	3.473	1.971	0.181	0.114
400	0.057	0.038	3.523	1.569	3.57	0.643	2.96	0.432	2.855	0.605	3.219	1.442	0.113	0.099
800	0.049	0.038	3.263	0.985	3.185	0.304	2.421	0.216	2.733	0.918	2.794	0.963	0.094	0.078
1600	0.05	0.036	3.111	0.913	2.741	0.219	2.122	0.147	2.551	0.228	2.728	0.78	0.072	0.064
3200	0.04	0.036	2.749	0.591	2.211	0.131	1.746	0.09	2.031	0.14	2.23	0.411	0.057	0.051
6400	0.039	0.037	2.29	0.392	1.97	0.087	1.256	0.063	1.744	0.085	1.874	0.271	0.061	0.146
12800	0.039	0.037	1.715	0.282	1.147	0.066	0.833	0.049	1.369	0.242	1.363	0.144	0.04	0.033
25600	0.037	0.037	1.073	0.095	0.651	0.043	0.489	0.048	0.877	0.044	0.935	0.088	0.037	0.038
51200	0.038	0.037	0.693	0.082	0.411	0.042	0.304	0.039	0.568	0.043	0.563	0.056	0.037	0.037
102400	0.037	0.036	0.467	0.051	0.229	0.039	0.169	0.035	0.345	0.039	0.329	0.044	0.035	0.037
204800	0.036	0.037	0.217	0.043	0.133	0.036	0.1	0.037	0.199	0.038	0.195	0.04	0.035	0.037

© 2014 by the authors; licensee MDPI, Basel, Switzerland. This article is an open access article distributed under the terms and conditions of the Creative Commons Attribution license (http://creativecommons.org/licenses/by/4.0/).
